# Disorganization of intercalated discs in dilated cardiomyopathy

**DOI:** 10.1038/s41598-021-90502-1

**Published:** 2021-06-04

**Authors:** Yukinobu Ito, Makoto Yoshida, Hirotake Masuda, Daichi Maeda, Yukitsugu Kudo-Asabe, Michinobu Umakoshi, Hiroshi Nanjo, Akiteru Goto

**Affiliations:** 1 Department of Cellular and Organ Pathology, Graduate School of Medicine, Akita University, 1-1-1 Hondo, Akita, Akita 010-8543 Japan; 2 Akita Karyology and Histology Research Center, Akita, Japan; 3 Department of Clinical Genomics, Graduate School of Medicine, Osaka University, Osaka, Japan; 4 Department of Clinical Pathology, Akita University Hospital, Akita, Japan

**Keywords:** Cardiomyopathies, Heart failure

## Abstract

Dilated cardiomyopathy (DCM) is a primary myocardial disease, the pathology of which is left ventricular or biventricular dilation and impaired myocardial contractility. The clinical and pathological diagnosis of DCM is difficult, and other cardiac diseases must be ruled out. Several studies have reported pathological findings that are characteristic of DCM, including cardiomyocyte atrophy, nuclear pleomorphism, and interstitial fibrosis, but none of these findings are DCM-specific. In this study, we examined the morphological differences in the intercalated discs (ICDs) between three groups of patients, a DCM group, a chronic heart failure group, and a control group. A total of 22 autopsy cases, including five DCM cases, nine CHF cases and eight control cases, were retrieved from the archives of the Department of Pathology at Akita University, Japan. The morphological differences were examined using multiple methods: macroscopic examination, light microscopy, immunohistochemistry, electron microscopy, and gene expression analyses. We observed disorganized ICDs, clearly illustrated by N-cadherin immunostaining in the DCM group. “Reduction of N-cadherin immunostaining intensity” and “ICD scattering” was DCM-specific. The results suggest that disorganized ICDs contribute to the development of DCM, and that N-cadherin immunostaining is useful for determining the presence of disorganized ICDs and for the pathological diagnosis of DCM.

## Introduction

The heart has the ability to adapt to an increase or decrease in load, such as volume overload or pressure overload, and it compensates for these changes through dilation and hypertrophy^1–3^. Chronic heart failure (CHF) and DCM are conditions in which the heart cannot adapt to overload and undergoes irreversible remodeling^4^. Heart enlargement occurs due to ventricular remodeling, in which the ventricle wall becomes thinner over time. The detailed mechanism underlying this remodeling has not been elucidated but may involve a change in myocardial sheet structure^5,6^.

DCM is a myocardial disease characterized by left ventricular or biventricular dilation and impaired myocardial contractility^7^. Patients with DCM typically exhibit ventricular enlargement and subsequent cardiac dysfunction, which can be accompanied by arrhythmia, thromboembolism, or sudden death^8,9^. The most common etiology of dilated cardiomyopathy (DCM) is idiopathic, and reported secondary causes of DCM include heredity, infection, non-infectious inflammation, poisoning (alcohol, etc.), trauma, and endocrine and metabolic disorders^10–12^. The incidence of DCM is reported to be 5–7 cases per 100 000 people per year^13^. Previous reports revealed that the prevalence of DCM was 36.5 cases per 100,000 people per year^14^. DCM is associated with contractile impairment and changes in cardiomyocyte shape^15^. Cytoskeletal abnormalities may be a common feature of this disease^15^. However, the pathogenesis of DCM remains unclear in most cases^12^.

Histopathologically, it is difficult to distinguish between DCM and CHF with marked cardiac dilation. The characteristic features of DCM include irregular cardiomyocyte hypertrophy, cardiomyocyte elongation, nuclear pleomorphism, diffuse interstitial fibrosis, and myofibrillar loss^16^. However, some of these findings are occasionally encountered in dilated hearts resulting from cardiac diseases other than DCM. To achieve more accurate pathological diagnosis, it would be useful to identify histopathological findings more specific to DCM.

N-cadherin, also known as Cadherin-2 (CDH2) or neural cadherin (NCAD), is encoded by the *CDH2* gene. N-cadherin is predominantly expressed in myocardial cells, especially in intercalated discs (ICDs)^17,18^. Myocardial cells are joined together mechanically and electrically by N-cadherin–mediated adherens junctions, which also provide the anchor points for the cytoskeleton within the cytoplasm, thereby maintaining the structural integrity and polarity of the tissue in the adult organism^17,19,20^. The ability of myocardial cells to adapt to physiological changes may involve the formation of appropriate junctions by which cells adhere to and communicate with each other^17^. Changes in expression and distribution of N-cadherin are commonly observed during heart development^18^.

Previously, using a rabbit AV shunt model, we conducted a morphological observation study of cardiomyocytes and ICDs in compensatory heart enlargement due to volume overload^21^. When volume load was applied to the heart, sarcomere formation occurred at the ICDs^21^. In the process, we noticed that very few studies have described the morphological changes of ICDs in dilated remodeling in the compensated and decompensated phases in human heart samples. Consequently, it remains unclear how the ICD morphology differs between normal heart, CHF, and DCM. In this study, we examined the morphological differences in ICDs among these three groups using multiple modalities: macroscopic findings, light microscopy, immunohistochemistry, electron microscopy, and gene expression analyses. Our results reveal that ICD disorganization is a characteristic of DCM and demonstrate that N-cadherin immunohistochemistry is useful for identifying this feature.

## Results

### Clinical features and follow-up information

The clinical diagnosis and autopsy findings of each case are shown in Table 1. The average ages of the patients in each group were as follows: control, 65 years; CHF, 65 years; and DCM, 50.4 years. The patients in the DCM group were younger than those in the other two groups. In addition, all patients with DCM were male. The DCM patients had no relevant family history, i.e., they were sporadic cases. Average heart weights were as follows: control, 375.0 g; CHF, 583.3 g; and DCM, 537.0 g. Heart weight did not significantly differ between the CHF and DCM groups. The average EF values were as follows: control, 71.7%; CHF 50.1%; and DCM, 18.6%. In the DCM group, EF was significantly reduced (P < 0.05), and EF decreased as DCM got worse. No correlation between heart weight and EF was observed in the control and CHF groups. In all cases, the EF was inversely correlated with the estimated LV volume.

**Table 1:** Clinical diagnosis and autopsy findings of each case.

Group	Age	Sex	Clinical diagnosis	Heart weight (g)	Ejection fraction (%)	LV wall thickness (mm)	Estimated LV volume (mm^2^)	Cardiomyocyte length (mm)	ICD width (mm)	ICD scattering (mm)
Control	68	M	Lung cancer	390	–	13	311.0	129.8	2.74	8.79
	65	F	Chronic myelogenous leukemia	350	–	13	216.8	99.7	2.94	8.05
	63	M	Prostatic cancer	415	–	12	293.7	105.1	2.75	7.27
	81	F	Subarachnoid hemorrhage	355	79.9	15	198.7	96.5	2.56	4.74
	26	F	Malnutrition	220	64.3	13	716.3	99.9	1.31	3.65
	71	M	Gastric cancer	410	–	16	117.8	88.1	2.10	3.24
	71	M	Gallbladder cancer, liver failure	345	–	15	223.8	86.5	2.78	6.06
	75	M	Gastric cancer	515	71.0	16	238.8	93.2	3.42	6.79
Chronic heart failure	53	F	CHF, MVR, Myasthenia gravis	285	24.3	5	633.0	106.5	4.94	12.14
	88	F	CHF, Old myocardial infarction	400	60.4	8	1044.6	87.8	5.14	8.86
	78	M	CHF, Aortic valve stenosis	820	61.8	12	235.6	99.5	4.12	7.65
	85	M	CHF	610	48.6	17	1283.3	104.4	3.96	7.50
	63	M	CHF, Acute myocardial infarction	620	–	18	993.5	98.4	3.15	5.75
	21	M	CHF, Malignant lymphoma	330	51.3	12	714.7	94.1	1.15	3.14
	84	M	CHF, Coronary arteriovenous fistula	815	54.1	13	112.3	87.5	2.60	6.06
	62	M	CHF, Acute myocardial infarction	775	50.0	15	1162.4	127.1	4.70	9.71
	51	M	CHF, Acute myeloid leukemia	595	–	11	1698.0	101.3	2.91	9.00
Dilated cardiomyopathy	28	M	DCM	465	20.0	11	888.3	108.5	6.39	17.00
	64	M	DCM	430	28.0	4	2186.5	113.9	5.67	25.60
	28	M	DCM	410	–	7	1873.2	122.2	5.28	25.00
	72	M	Combined valvular disease, CHF	730	13.0	7	4472.8	133.5	5.81	18.70
	60	M	DCM	650	13.4	7	2552.5	115.9	4.76	23.56

Twenty-one autopsy cases were retrieved from the archives of the Department of Pathology at Akita University, Japan. We classified these cases into three groups (control, CHF, and DCM) based on the clinical diagnoses and histological findings. The average ages of the patients in each group were as follows: control, 65.0 years; CHF, 65.0 years; and DCM, 50.4 years. The patients in the DCM group were younger than those in the other two groups; in addition, all DCM patients were male. Average heart weights in each group were as follows: control, 375.0 g; CHF, 583.3 g; and DCM, 537.0 g.

In each case, the cardiomyocyte length, ICD width, and ICD scattering were all inversely correlated with the EF and positively correlated with the estimated LV volume.

*CHF* chronic heart failure, *DCM* dilated cardiomyopathy, *ICD* intercalated disc, *LV* left ventricle, *MVR* mitral valve regurgitation.

### Differences in macroscopic findings among the three groups

#### DCM versus control

**Figure 1:**
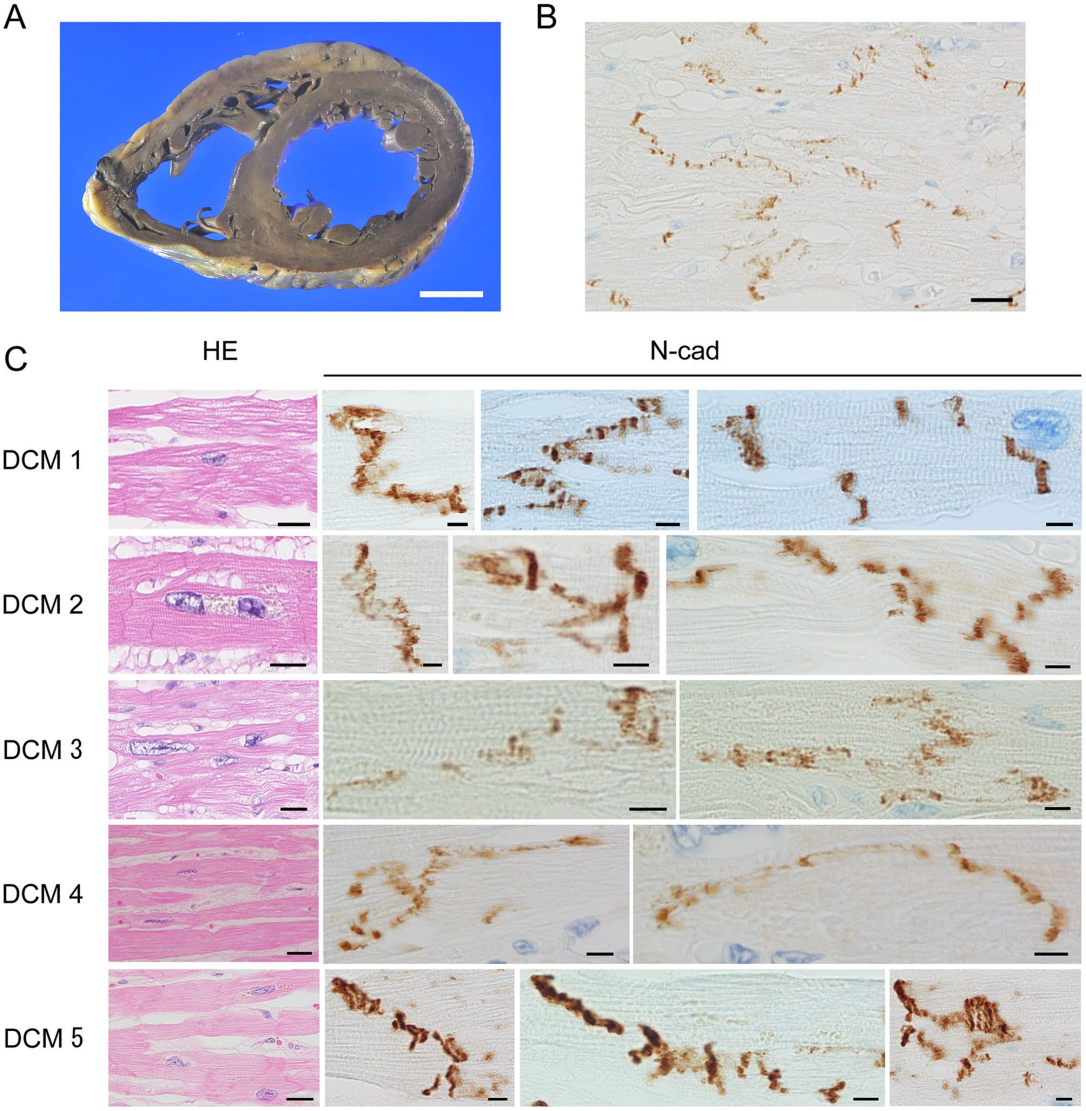
Pathological and immunohistochemical findings in dilated cardiomyopathy. (**A**) Macroscopically, we observed severe dilation of the bilateral ventricles. LV wall thickness in the DCM group was relatively uniform, but the wall was thinner in DCM than in the other two groups. (**B**) ICDs were partially but not clearly observed, and cardiomyocyte units were unclear. (N-cadherin immunostaining; scale bar, 20 µm; original magnification, × 400). (**C**) Characteristic findings of DCM included cardiomyocyte atrophy, nuclear pleomorphism, and interstitial fibrosis (**H**–**E** staining; scale bar, 20 µm; original magnification, × 400). Immunohistochemistry revealed that N-cadherin immunostaining was lower in the DCM group than in the CHF and control groups (N-cadherin immunostaining; scale bar, 5 µm; original magnification, × 1000). ICD width was approximately 2–8 sarcomeres and was significantly elongated in the long-axis direction of the cardiomyocytes. The ICDs were scattered, had a stepwise shape, and were highly curved.

Macroscopically, in the DCM group, the lumen of the LV was remarkably dilated, and the wall was thin in comparison with the control group (Fig. 1A). The LV wall was uniformly thin, and irregular fibrosis was observed on the entire LV wall. Average LV wall thickness was 14.1 mm in the control group, 12.3 mm in the CHF group, and 7.2 mm in the DCM group (Table 1). Estimated LV volume was highest in the DCM group; average left ventricle (LV) volumes were as follows: control, 289.6 mm^2^; CHF, 875.3 mm^2^; and DCM, 2394.7 mm^2^. On the other hand, in the control group, the LV wall had a constant thickness, as in the DCM group, but no fibrosis was observed in the coronal section of the heart (Fig. 1A).

#### DCM versus CHF

The lumen of the LV was dilated to a greater extent in the DCM group than in the CHF group (Figs. 1A, 3A). The estimated LV volume was significantly higher in the DCM group than in the CHF group (Table 1), and the LV wall was significantly thinner in the DCM group than in the CHF group (Table 1). However, these values varied widely in the CHF group: some cases were as dilated as in DCM, whereas others were similar to the control group.

#### CHF versus control

**Figure 2:**
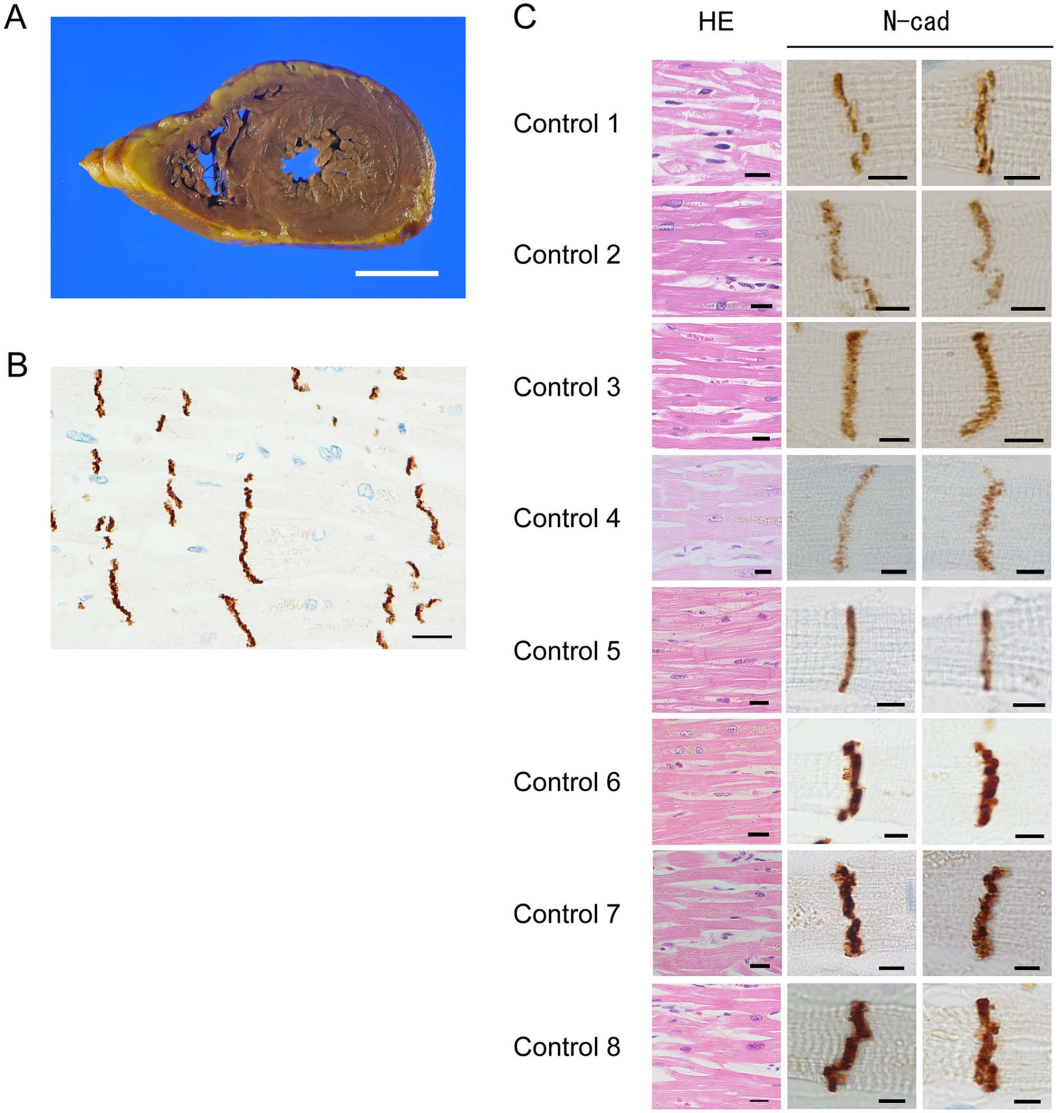
Pathological and immunohistochemical findings in control cases. (**A**) Macroscopically, we observed no dilation of the bilateral ventricles. The LV wall had a uniform thickness. (**B**) ICDs could be clearly observed, and cardiomyocyte units were clear. (N-cadherin immunostaining; scale bar, 20 µm; original magnification, × 400). (**C**) Histologically, ICDs were observed between cardiomyocytes (**H**–**E** staining; scale bar, 20 µm; original magnification, × 400). Each cardiomyocyte could be clearly distinguished. Immunohistochemistry for N-cadherin revealed positive staining at ICDs, and ICDs were thin and flat. (N-cadherin immunostaining; scale bar, 5 µm; original magnification, × 1000). ICD width was within two sarcomeres.

**Figure 3:**
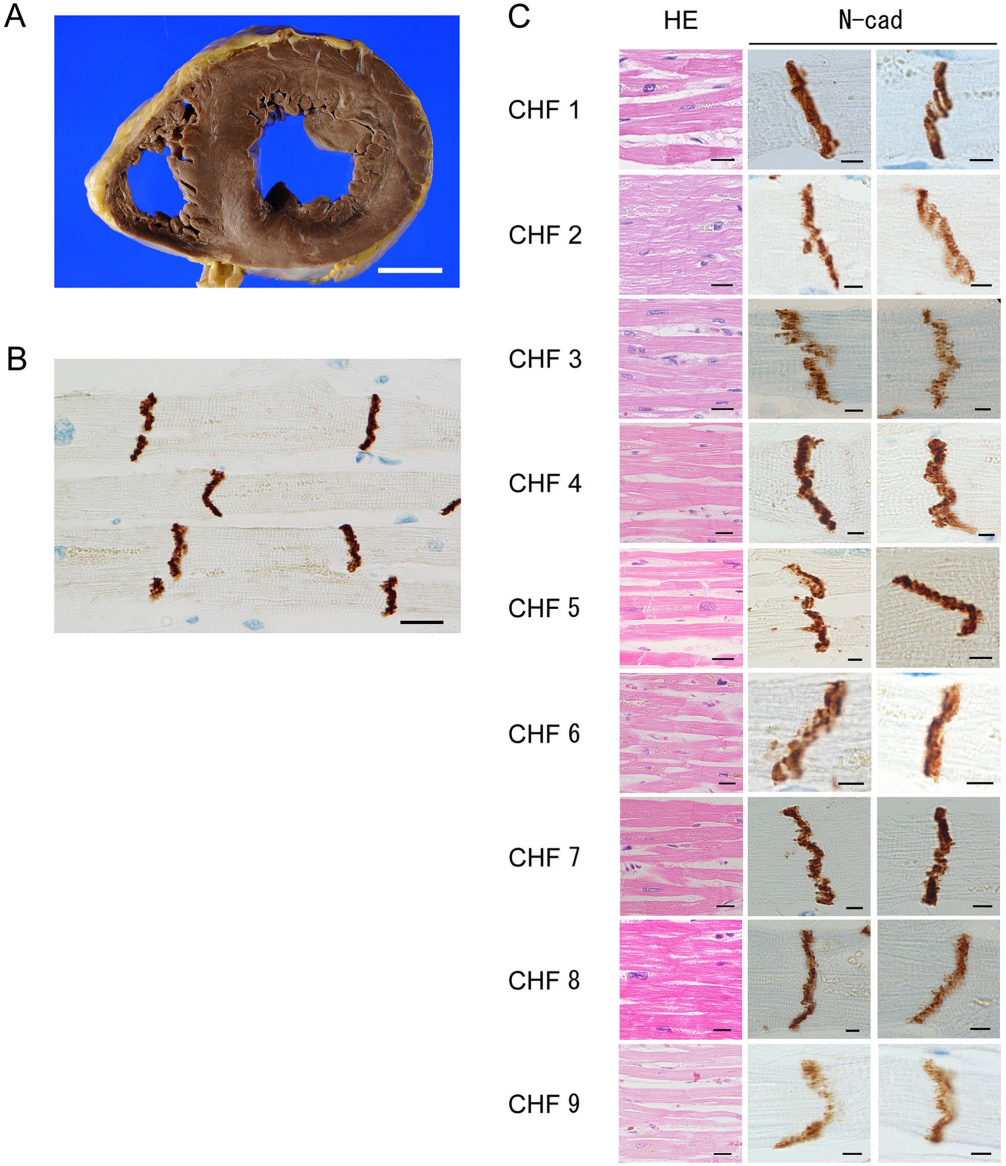
Pathological and immunohistochemical findings in cases of chronic heart failure. (**A**) Macroscopically, we observed mild-to-moderate dilation of the bilateral ventricles. The LV wall was thinner than in the control group. Depending on the cause of CHF, such as acute myocardial infarction, thickness was sometimes uneven. (**B**) ICDs could be seen relatively clearly, and the units of cardiomyocytes were clear. (N-cadherin immunostaining; scale bar, 20 µm; original magnification, × 400). The width of the ICDs was slightly wider in the CHF group than in the control group. (**C**) Histologically, ICDs were observed between cardiomyocytes, but their structures were somewhat irregular and hard to see (H-E staining; scale bar, 20 µm; original magnification, × 400). Immunohistochemistry for N-cadherin revealed that the width of the ICDs was approximately two to six sarcomeres, and was wider than in the control group (N-cadherin immunostaining; scale bar, 5 µm; original magnification, × 1000). The ICDs had a stepwise appearance and were crooked.

The lumen of the LV was more dilated in the CHF group than in the control group (Figs. 2A, 3A). The LV wall was thinner in the CHF group than in the control group, whereas LV volume as significantly higher in the CHF group. However, the degrees of LV dilation and fibrosis, LV wall thickness, and LV volume varied widely among the cases.

### The differences in histopathological findings among the three groups

In general, histological findings of DCM hearts included cardiomyocyte hypertrophy, cardiomyocyte elongation, nuclear pleomorphism, diffuse interstitial fibrosis, and myofibrillar loss. All cases in the DCM group shared these findings. On the other hand, some cases in the CHF group did not exhibit these findings, whereas others did. Myofibrillar disarray, vacuolization, granuloma, and inflammatory cell infiltration was not observed in any of the three groups.

#### DCM versus control

Histologically, in the DCM group, cardiomyocytes were thinner than in the control group, and diffuse fibrosis was observed between cardiomyocyte bundles (Fisg. 1B, C HE). ICDs were partially but not clearly visible. Consequently, the units of cardiomyocytes were unclear. In the control group, we observed no fibrosis between cardiomyocyte bundles (Fig. 2B, C HE), and ICDs were relatively clearly visible between the cardiomyocytes.

#### DCM versus CHF

In the CHF group, ICDs were visible between cardiomyocytes, but their structures were irregular and difficult to see (Fig. 3B, C HE). In some cases in the CHF group, the ICDs were slightly irregular, as in DCM. Moreover, in some cases in the CHF group, fibrosis was present in the cardiomyocyte bundles, but to widely varying degrees.

#### CHF versus control

Histologically, there is no significant difference between the CHF and control group.

### Differences in immunohistochemical findings of N-cadherin immunostaining among the three groups

#### DCM versus control

In the DCM group, immunohistochemical staining for N-cadherin revealed that the ICDs were disorganized and wider than in the control and CHF groups (Fig. 1B, C N-cad). The ICDs were significantly elongated in the long-axis direction of the cardiomyocytes. Pathological findings in the ICDs included duplication, curving, meandering, zigzagging, and scattering. Moreover, N-cadherin immunostaining intensity in the DCM group was lower than that in the control group, and ICDs were obscure. By contrast, in the control group, the ICDs were clearly stained by N-cadherin antibody and were almost straight (Fig. 2B, C N-cad).

#### DCM versus CHF

ICDs were wider in the DCM group than in the CHF group (Figs. 1B, 3C N-cad), and were spatially widely scattered in cardiomyocytes. Also, N-cadherin immunostaining intensity in the DCM group was lower than in the CHF group. In some cases in the CHF group, ICDs were as wide as in DCM.

#### CHF versus control

ICD width and N-cadherin immunostaining intensity did not significantly differ between the CHF and control groups (Figs. 2C, 3C N-cad). However, in the CHF group, the ICDs were a bit wider than in the control group, and had a stepwise appearance and slightly crooked. ICD scattering was not observed in both groups.

### Transmission electron microscopy findings of ICDs in each group

#### DCM versus control

**Figure 4:**
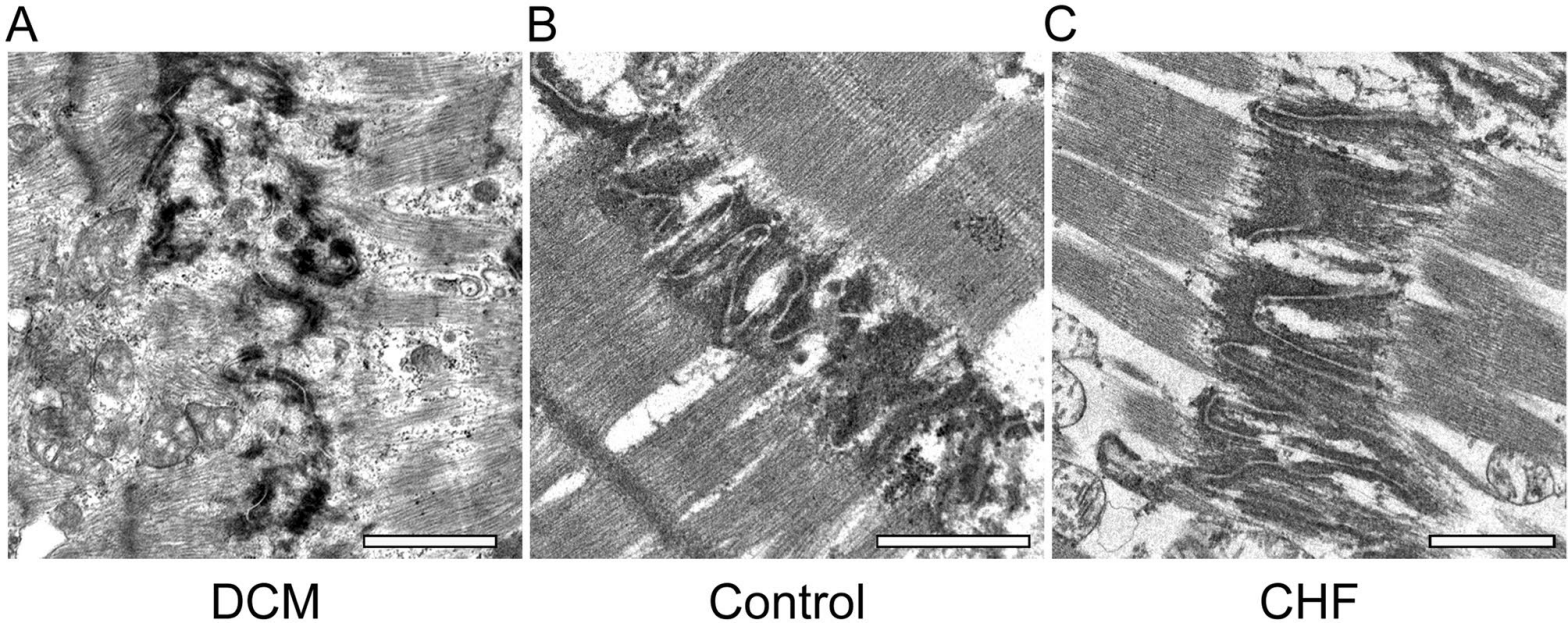
Transmission electron microscopy findings of intercalated discs in each group. (**A**) In the DCM group, ICDs were deteriorated and disorganized, the interdigitation of ICDs was disrupted throughout the tissue, and the junctions between cardiomyocytes and ICDs were obscured. Sarcomeres were arranged in a complex manner (scale bar, 1 μm; original magnification, × 6000). (**B**) In the control group, ICD ultrastructure was preserved. The finger-like folds of ICDs, called interdigitation, repeated at regular intervals (scale bar, 1 μm; original magnification, × 6000). (**C**) In the CHF group, ICDs were wider than in the control group, about two sarcomere widths. However, ICD ultrastructure as preserved. Interdigitation of ICDs was retained (scale bar, 1 μm; original magnification, × 6000).

In the DCM group, ICD ultrastructure was deteriorated and disorganized (Fig. 4A); specifically, the interdigitation of ICDs was disrupted throughout the tissue, the junctions between cardiomyocytes and ICDs were obscured, and the sarcomeres were arranged in a complex manner. On the other hand, in the control group, the interdigitation repeated at regular intervals (Fig. 4B), and the junctions between cardiomyocytes and ICDs were clearly visible. The ICDs and sarcomeres of the control group were not deteriorated.

#### DCM versus CHF

ICD width was greater in the DCM group than in the CHF group (Fig. 4A, B). Some of the sarcomeres had disappeared, and the remaining ones were arranged in a complex manner. In the CHF group, the ICD ultrastructure was preserved in the CHF group, and interdigitation of ICDs was also retained.

#### CHF versus control

In the CHF group, ICDs were bit wider than in the control group, spanning about two sarcomeres (Fig. 4C). However, the ICD ultrastructure was preserved in both groups, and interdigitation of ICDs was also retained.

### Measurement of cardiomyocyte length, ICD width, and scattering in each group

**Figure 5:**
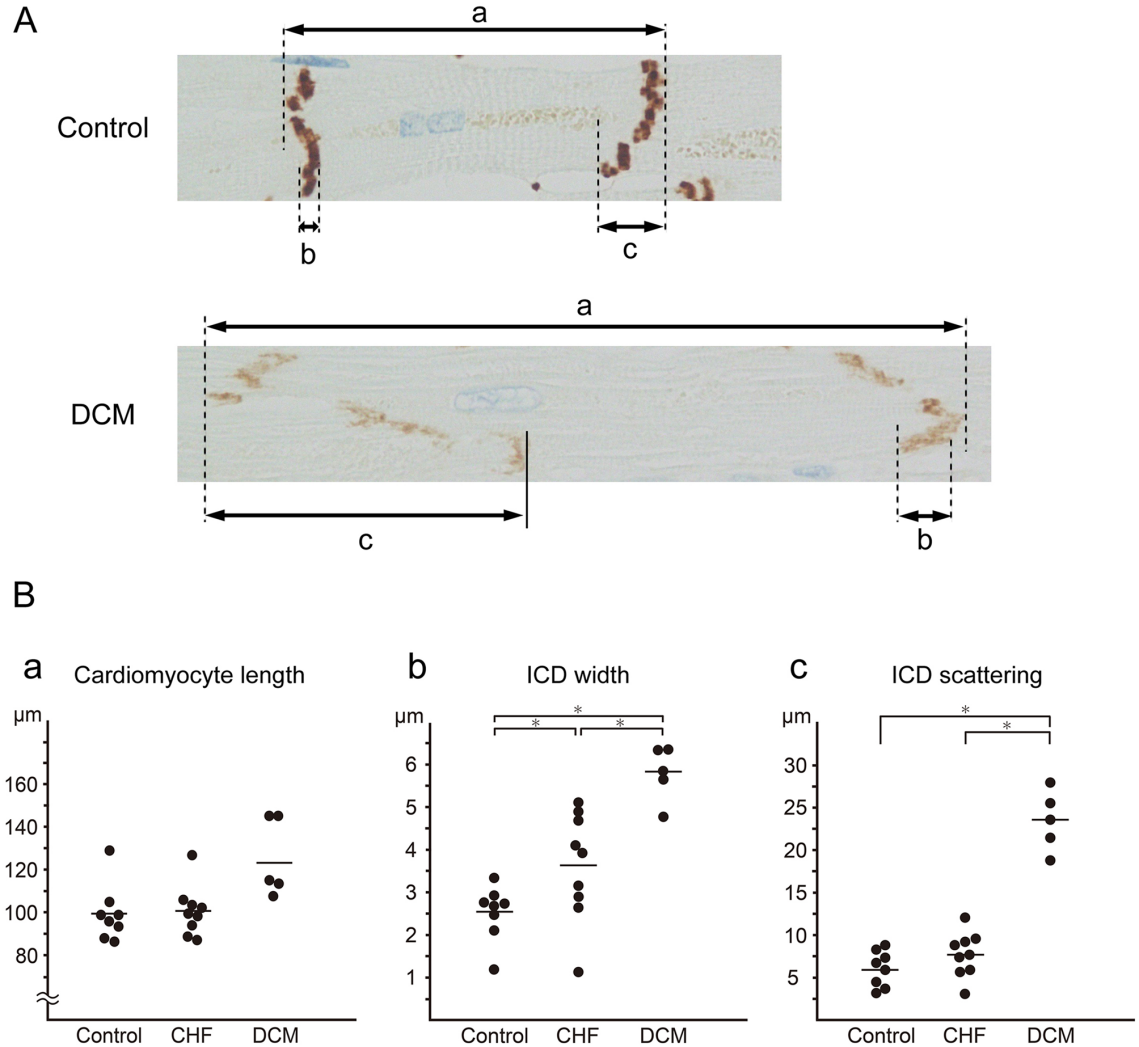
Cardiomyocyte length, ICD width, and ICD scattering in each group. (**A**) Morphology of cardiomyocytes and ICDs in each group was observed in N-cadherin–stained specimens. Cardiomyocyte length (a), ICD width (b) and ICD scattering (c) were measured. (**B**) Cardiomyocyte length was significantly greater in the DCM group than in the control groups (a). ICD width was significantly greater in the DCM group than in the CHF and control groups (b), and differed significantly between the CHF group and the control group. The variation in the CHF cases was large. ICDs were more scattered in the DCM group than in the CHF and control groups (c). Statistical significance of differences between groups was determined using the Mann–Whitney U test: *P < 0.05.

The result of pathological measurement was shown in Table 1. Average cardiomyocyte lengths were as follows: control, 99.85 μm; CHF, 100.7 μm; and DCM, 123.39 μm (Fig. 5B-a). We observed no significant differences among the groups. Average ICD widths were as follows: control, 2.56 μm; CHF, 3.63 μm; and DCM, 5.80 μm (Fig. 5B-b); the widths differed significantly between the control and DCM groups (P < 0.05). In the CHF group, we observed variation from values ranging near those in the control group to near those in the DCM group. Average ICD scattering was as follows: control, 6.07 μm; CHF, 7.76 μm; and DCM, 23.50 μm (Fig. 5B-c). The scattering differed significantly between the DCM group and the control and CHF groups (P < 0.05).

In each case, the cardiomyocyte length, ICD width, and ICD scattering were all inversely correlated with the EF and positively correlated with the estimated LV volume.

From the results of immunohistochemical findings and ICD measurement, we defined these findings as “Reduction of immunostaining intensity”, “ICD widening” and “ICD widening”, and summarized in Table 2. “Reduction of immunostaining intensity” and “ICD scattering” was observed exclusively in the DCM group, whereas “ICD widening” was not group-specific.

**Table 2:** Summary of pathological findings in three groups.

	Control	CHF	DCM
**Histological findings**			
Cardiomyocyte hypertrophy	−	±	+
Cardiomyocyte elongation	−	±	+
Nuclear pleomorphism	−	±	+
Diffuse interstitial fibrosis	−	±	+
Myofibrillar loss	−	±	+
Myofibrillar disarray	−	−	−
Vacuolization	−	−	−
Granulomas	−	−	−
Inflammatory cell infiltration	−	−	−
**Immunohistochemical findings of N-cadherin staining**			
Reduction of stainability	−	−	+
ICD widening	−	±	+
ICD scattering	−	−	+

Pathological and immunohistochemical findings from the control, CHF, and DCM groups are summarized in this table. Deterioration of N-cadherin immunostaining was defined as a reduction in immunostaining relative to the control group. Moreover, ICD widening was defined as a width of 4 µm or more, and ICD scattering as 15 µm or more. The meanings of the symbols used in this table are as follows: (−): This finding is absent in most cases. (±): This finding is present in some cases. (+): This finding is present in most cases.

*CHF* chronic heart failure, *DCM* dilated cardiomyopathy, *ICD* intercalated disc.

### Gene expression analysis of ICD-associated proteins by quantitative real-time PCR

**Figure 6:**
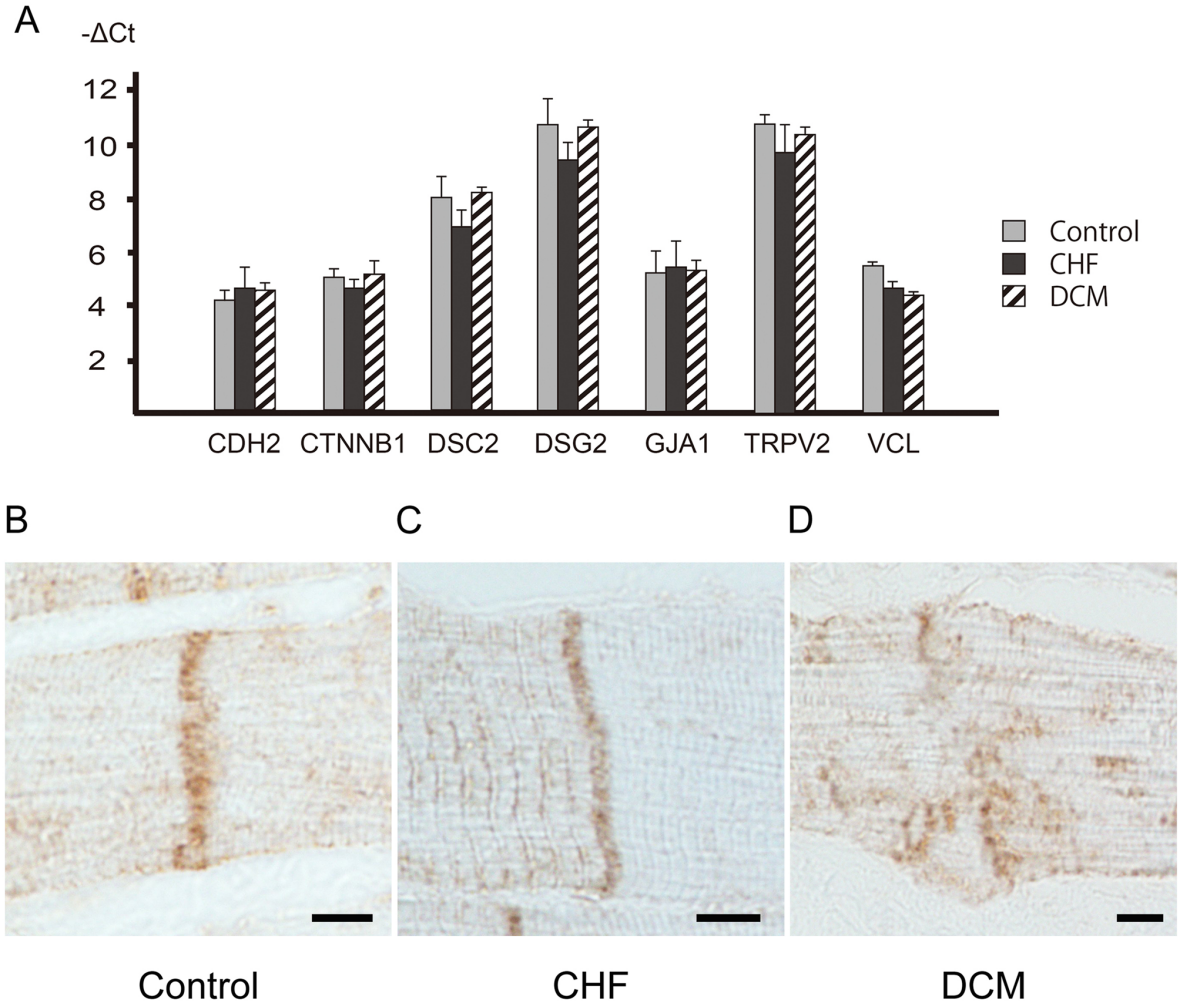
Expression of genes associated with intercalated discs, and immunohistochemical staining of vinculin. (**A**) Expression of *CDH2, CTNNB1, DSC2, DSG2, GJA1, TRPV2,* and *VCL* genes in the LV samples from the three groups was evaluated by TaqMan quantitative real-time PCR. The gene expressions of ICD-associated genes, such as *CDH2, CTNNB1, DSC2, DSG2, GJA1, TRPV2,* and *VCL*, did not differ significantly between the DCM group and the CHF and control groups. The statistical significance of differences between groups was determined by the Mann–Whitney U test. (**B**) In the positive control group, immunohistochemistry revealed positive staining for vinculin in the ICD, myocardial cell membrane, and sarcomere. Immunostaining of vinculin was weaker than that of N-cadherin, and the ICD was hard to recognize. The ICD was lightly stained (vinculin immunostaining; scale bar, 5 µm; original magnification, × 1000). (**C**) As in the control group, the ICD stained lightly for vinculin. The myocardial cell membrane and sarcomere were also stained (vinculin immunostaining; scale bar, 5 µm; original magnification, × 1000). (**D**) In the DCM group, vinculin immunostaining was even weaker than in the CHF group, and ICDs were difficult to recognize. ICDs were deteriorated and disorganized (vinculin immunostaining; scale bar, 5 µm; original magnification, × 1000).

We performed quantitative real-time PCR to analyze the expression of *CDH2, CTNNB1, DSC2, DSG2, GJA1, TRPV2,* and *VCL* in each group. The ∆Ct values for *CDH*2, *CTNNB1*, *DSC2*, *DSG2*, *GJA1, TRPV2*, and *VCL* are shown in Fig. 6. The gene expression of these ICD-associated proteins, including *CDH2, CTNNB1, DSC2, DSG2, GJA1, TRPV2* and *VCL*, did not differ significantly among the groups.

### Immunohistochemical findings of vinculin in each group

In the control group, vinculin immunohistochemistry stained the ICD, myocardial cell membrane, and sarcomere (Fig. 6B). The immunostaining of vinculin was also weaker than that of N-cadherin; consequently, it was difficult to recognize the ICD. Similar findings were obtained in the CHF group (Fig. 6C). In the DCM group, the ICD was almost entirely obscure for vinculin immunostaining (Fig. 6D).

## Discussion

Because N-cadherin is generally localized in ICDs, it stains as a single well-ordered band between cardiomyocytes. Our findings revealed that in the DCM group, ICDs were stretched, fragmented, and degraded. Consequently, we hypothesized that N-cadherin immunostaining intensity of ICDs was reduced. This reduction in N-cadherin immunostaining intensity was not observed in the CHF or control groups. In general, the dilated phase of HCM is macroscopically similar to DCM; however, in such cases, we observed no change in the N-cadherin immunostaining intensity in the ICDs (see Supplementary Fig. S1). This finding was specific to DCM. DCM can be induced by knockout of the N-cadherin gene^22,23^. Moreover, germline mutations have been reported in genes coding for ICD-associated membrane proteins in some DCM cases^24^. Our study supports these previous findings and suggests that DCM is a disorder of the ICDs. Moreover, our TEM observations revealed that in the DCM group, interdigitation of ICD ultrastructure was deteriorated and disorganized, and the junctions between cardiomyocytes and ICDs were obscured. These findings may be associated with the reduction of N-cadherin immunostaining intensity.

We then measured cardiomyocyte length, ICD width, and ICD scattering. ICD scattering differed significantly between the DCM group and the CHF and control groups. We concluded that ICD scattering was a characteristic finding in DCM that would be useful for diagnosing this condition. In CHF, ICD width varied widely among cases, with some ICDs as wide as those in DCM and others only as wide as those in the control group. An inverse correlation was observed between the ICD width and EF, thus suggesting that ICD width gradually increases as heart failure progresses. Furthermore, patients in their twenties tended to have lower ICD width in both the CHF and control groups, implying that this factor may be related to age. Accordingly, we concluded that ICD width would not be useful for diagnosing DCM. In addition, we noted no significant differences in the cardiomyocyte length between the DCM group and the other groups. In a previous study, we reported that control cardiomyocyte length was 130.1 ± 32.6 µm^25^, and this result was consistent with the finding of our present study. Accordingly, we concluded that cardiomyocyte length would not be useful for diagnosing DCM.

There were three limitations associated with this study. First, the number of cases to be studied was small. In this study, we analyzed autopsy cases. Although it would have been easy to increase the number of cases in the normal group, the numbers of CHF and DCM cases were limited. In particular, DCM cases were extremely rare, and further increasing the number of cases analyzed was not possible at the time we conducted this study. Although we confirmed statistically significant differences and correlations in this study, there are still some uncertainties regarding the statistical analyses due to the limited number of samples. We hope that this analysis will continue and that researchers around the world will continue to verify our findings. Second, it was difficult to define DCM. In this study, we excluded secondary DCMs, such as ischemia, other types of cardiomyopathy, and drug-induced DCM. DCM is an exclusionary diagnosis, so its definition varies according to each clinician or researcher. Third, our method for measuring ICDs was also considered to be a limitation because our measurement method was that adjacent ICDs could not be clearly distinguished due to the collapse of ICDs. The ICDs were broken and stretched, and some were in contact with adjacent ICDs. In this study, we excluded cardiomyocytes in which the N-cadherin–stained area was spread to the adjacent cardiomyocytes, as well as ICDs that were difficult to measure. Hence, it is possible that the ICD width in our study was smaller than the actual ICD width. Similarly, ICD scattering may have been smaller than the actual values in the cases we examined.

Taken together, our findings suggest that reductions in N-cadherin immunostaining intensity and the ICD scattering are characteristic findings in DCM that could be useful for the diagnosis of DCM. In this study, there were two cases where the final diagnosis was changed using our findings, and these two cases were also included in this study. The first case was clinically suspected to have either adriamycin cardiomyopathy or DCM, but based on the pathological findings on autopsy, we deemed that this case should belong to the CHF group because there was little ICD scattering and ICDs with a reduced intensity of N-cadherin immunostaining. This case is presented in Supplementary Fig. S2. On the other hand, the second case was clinically diagnosed to be combined valvular disease and CHF, but the pathological findings on autopsy, such as the presence of ICD scattering and decreases in N-cadherin immunostaining intensity, were consistent with a diagnosis of DCM. This case is presented in Supplementary Fig. S3. Until now, few pathological findings have been reported to be specific to DCM, and thus it has been difficult to arrive at a pathological diagnosis. Our study showing that reduction in N-cadherin immunostaining intensity and the ICD scattering are characteristic features of DCM should make it possible to arrive at a pathologically definitive diagnosis of this disease in many patients.

ICDs consist of multiple proteins, including β-catenin, desmoglein 2, desmocollin 2, plakophilin, plakoglobin, connexin 43, and others (14). Maeda et al. reported that DCM is associated with deficiency of metavinculin, an isoform of vinculin found in cardiomyocytes^26^. Olson et al. revealed that vinculin and metavinculin play critical roles in cardiac structure and function, and that mutations in metavinculin may lead to DCM^27^. In cardiomyocytes, vinculin and metavinculin co-localize in ICDs, and both proteins are located at principal sites of contractile force transmission^27,28^. We analyzed the expression of ICD-associated genes, which revealed that *VCL* expression was lower in the DCM group than in the control group. However, in the statistical analysis, no significant difference was found in the expression of ICD-associated genes, including *VCL*. Also, immunohistochemical staining for vinculin was positive in both ICDs and sarcomeres, and it was difficult to clearly observe ICD deterioration and disorganization. Consequently, no difference was apparent between the groups, and it would be difficult to apply these findings to routine pathological diagnosis. We concluded that immunohistochemical staining of N-cadherin is more useful for diagnosis of DCM than immunostaining for vinculin.

From both morphological and molecular biological standpoints, the ICDs in the DCM group were deteriorated and disorganized. We hypothesized that the deterioration and disorganization of ICDs diminished N-cadherin immunostaining intensity. The ICD is very important for the exertion of cardiac contractile force^27^. Thus, DCM may suppress the cardiac contractile force via collapse of the ICD. However, the role of ICDs in this disease is not yet completely understood. Elucidation of the functions of ICDs is important for understanding heart diseases such as DCM.

## Materials and methods

### Case selection

A total of 22 autopsy cases were retrieved from the archives of the Department of Pathology at Akita University, Japan. Among them were five cases of DCM and nine cases of CHF; the remaining eight cases were assigned to the control group (Table 1). Patients in the control group had no other cardiac diseases such as coronary heart disease or valvular disease. In the CHF group, all cases were diagnosed with chronic heart failure with enlargement of the left ventricle. All patients of CHF group had presenting clinical symptoms of heart failure and a decreased ejection fraction (EF). This group includes 7 cases of heart failure due to valvular disease and ischemic heart disease, and two cases after chemotherapy. Specific cardiac disease cases, such as DCM, hypertrophic cardiomyopathy (HCM), Fabry's disease and etc., are excluded. In general, DCM is associated with clinically remarkable cardiac enlargement and reduced EF, as well as histopathologically irregular cardiomyocyte hypertrophy, cardiomyocyte elongation, nuclear pleomorphism, diffuse interstitial fibrosis, and myofibrillar loss. All cases in the DCM group were diagnosed clinically and pathologically using the general DCM diagnostic criteria. In this study, cases with secondary dilated cardiomyopathy, such as ischemia and drug-induced cases, were excluded.

The wall thickness and volume of the LV were measured based on the macroscopic findings at autopsy. The long and short diameters of the LV lumen at the short axis view were measured, and LV volume was estimated by the following formula:

$$\mathrm{Estimated \,LV \,volume }\left({\mathrm{mm}}^{2}\right)=\frac{\pi }{4}\times \left\{\mathrm{Long \,diameter }\left(\mathrm{mm}\right)\right\}\times \{\mathrm{Short \,diameter }\left(\mathrm{mm}\right)\}$$

Formalin-fixed, paraffin-embedded (FFPE) samples of all cases were collected and sectioned at a thickness of 1.5 μm. These sections were stained with hematoxylin–eosin (HE).

All procedures were performed in accordance with the ethical standards of the Helsinki Declaration. The study was approved by the institutional ethical committee of Akita University (approval number: 1246).

### Immunohistochemistry of N-cadherin and vinculin

FFPE samples of all cases were sectioned to a thickness of 4 μm, and the sections were subjected to immunohistochemical staining using anti-N-cadherin antibody (mouse monoclonal antibody [clone 6G11v; Dako Corp, Carpinteria, CA, USA) (dilution 1:50), and anti-vinculin antibody (mouse monoclonal antibody [clone 2B5A7]; Proteintech, Rosemont, IL, USA) (dilution 1:500). Immunostaining for N-cadherin and vinculin was performed on a Ventana Discovery XT autostainer (Ventana Medical Systems, Inc., Tucson, AZ, USA).

### Observation of ultrastructure in ICDs by transmission electron microscopy

A piece of LV from each case was harvested at autopsy and fixed in 2.5% glutaraldehyde solution. These pieces of tissue were cut into cubes (1 × 1 × 1 mm). Ultra-thin sections stained with lead citrate and uranyl acetate were observed by transmission electron microscopy (TEM) (H-7650; Hitachi, Ltd., Tokyo, Japan).

### Measurement protocol for ICDs and cardiomyocytes

Microscopic slide images were obtained using a NanoZoomer digital slide scanner (Hamamatsu Photonics, Hamamatsu, Japan). Using a whole slide image viewing software, “NDP view2” (Hamamatsu Photonics, Hamamatsu, Japan), we measured cardiomyocyte length (Fig. 5A-a), ICD width (Fig. 5A-b), and ICD scattering (Fig. 5A-c) in all cases. One hundred ICDs and cardiomyocytes were measured, and the average was calculated. Cardiomyocyte length was measured between adjacent ICDs, including the cardiomyocyte nucleus.

Based on the results of ICD measurements, we defined these findings as a “Reduction of immunostaining intensity”, “ICD widening” and “ICD widening”; they are summarized in Table 2. A “Reduction of immunostaining intensity” was defined as a clear reduction in N-cadherin immunostaining relative to the control group, “ICD widening” was defined as a width of ≥ 4 µm, and “ICD scattering” was defined as a width of ≥ 15 µm.

### Quantitative real-time polymerase chain reaction

The expression of ICD-related genes, which were previously reported, were evaluated by real-time PCR^17^. Expression of *CDH2* (Hs00983053_m1), *CTNNB1* (Hs00355045_m1), *DSC2* (Hs00951428_m1), *DSG2* (Hs00170071_m1), *GJA1* (Hs00748445_s1), *TRPV2* (Hs00901648_m1), and *VCL* (Hs00419715_m1) was evaluated by TaqMan quantitative real-time PCR assays (Thermo Fisher Scientific, Waltham, MA, USA) using RNA extracted from freshly frozen tissues. *GAPDH* (Hs02786624_g1) was used as an endogenous control to normalize target gene expression. Quantitative real-time PCR was performed on a 7900HT Fast Real-Time PCR System (Applied Biosystems, Life Technologies, Grand Island, NY, USA), and data were collected and analyzed using the SDS 2.3 software. All assays were performed in triplicate. Gene expression was quantified by calculating ∆Ct values, where Ct = threshold cycle and ∆Ct = Ct of target gene – Ct of *GAPDH*. Changes in expression were analyzed using DataAssist 2.0 (Applied Biosystems).

### Statistical analysis

Statistical analysis was performed with the GraphPad Prism 8 software program for Windows (GraphPad Software, San Diego, CA, USA). Data regarding cardiomyocyte length, ICD width, and ICD scattering are expressed as dot plots of the average from each case. Gene expression data obtained by RT-PCR are expressed as the average of each group. Considering the small sample size, statistical analyses were performed using the Mann–Whitney U test. P < 0.05 was considered to indicate statistical significance. The correlation analysis was performed using Spearman's rank correlation coefficient.

## Supplementary Information

Supplementary Information 1.
