# Supplementary material for: Disorganization of intercalated discs in dilated cardiomyopathy
Source: Sci Rep. 2021 Jun 4;11:11852. doi: 10.1038/s41598-021-90502-1 (PMC8178322; doi:10.1038/s41598-021-90502-1)
Supplement: Supplementary file 1 — Supplementary Information 1. [file 41598_2021_90502_MOESM1_ESM.docx]

**Supplementary Information for**

**Disorganization of Intercalated Discs in Dilated Cardiomyopathy**

Yukinobu Ito, Makoto Yoshida^*^, Hirotake Masuda, Daichi Maeda, Yukitsugu Kudo-Asabe, Michinobu Umakoshi, Hiroshi Nanjo, Akiteru Goto

***Corresponding author:**

Makoto Yoshida, MD, PhD

E-mail: myoshida@gipc.akita-u.ac.jp

Fig. S1


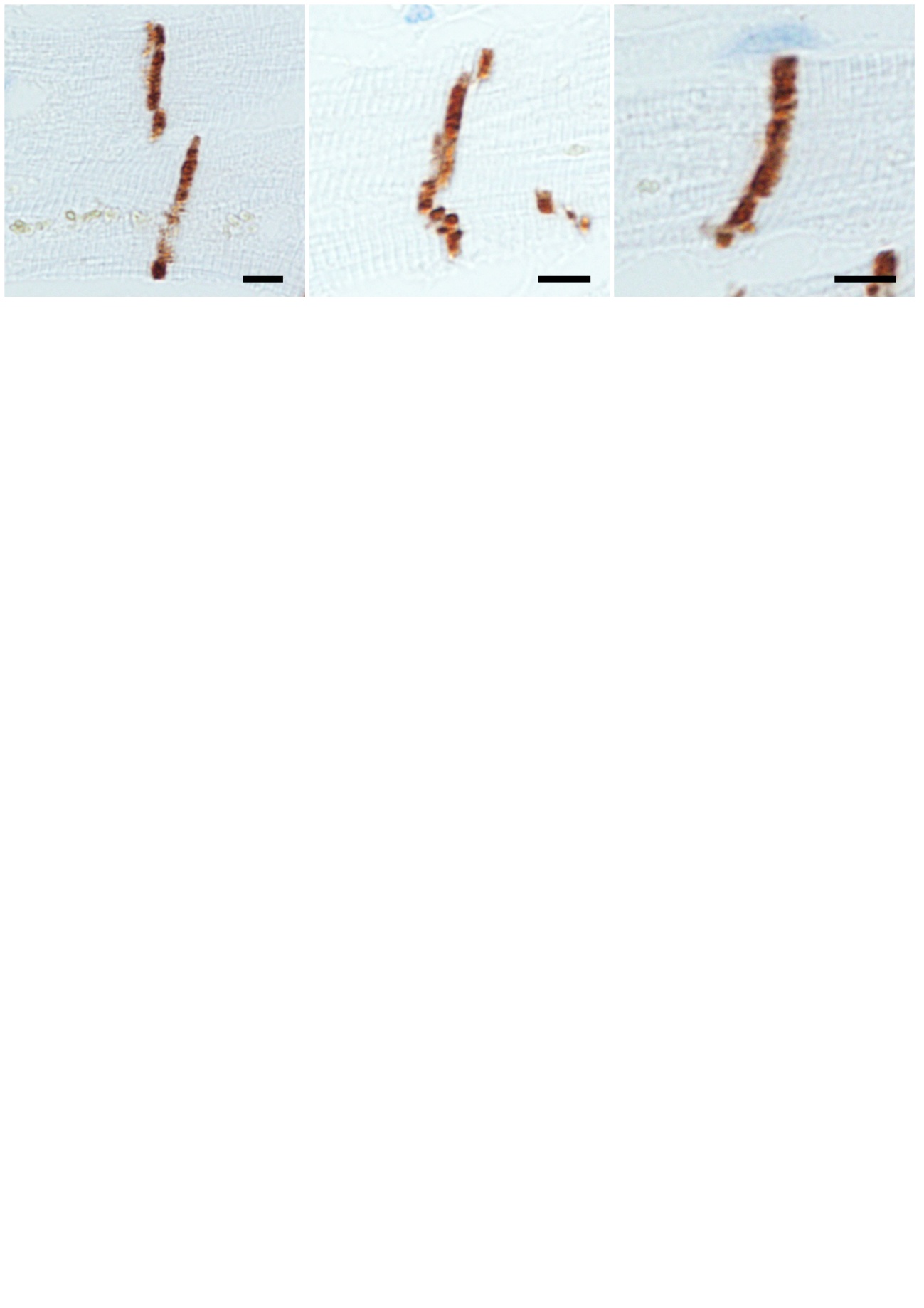


**Fig. S1 Immunohistochemical findings in Hypertrophic cardiomyopathy.**

Immunohistologically, in HCM, we observed no decrease in N-cadherin immunostaining (N-cadherin immunostaining; scale bar, 5 µm; original magnification, ×1000). In addition, ICDs were dyed in a band shape, with no evidence of deterioration or disorganization. Similar findings were obtained in the control and CHF groups.

Fig. S2


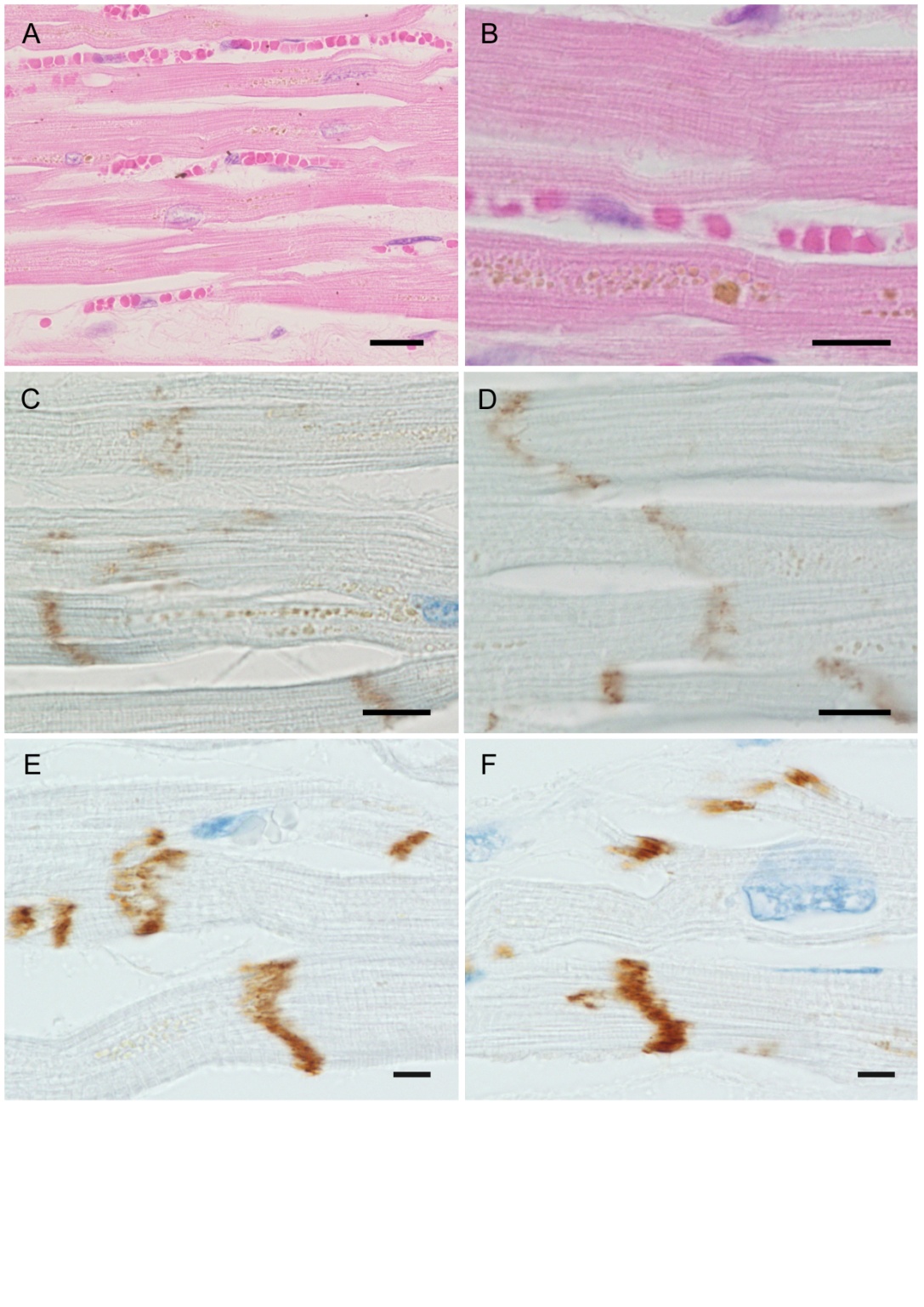


**Fig. S2 A case clinically diagnosed as adriamycin cardiomyopathy or DCM, was considered to be CHF based on the results of N-cadherin immunostaining and ICD scattering.**

This case was receiving adriamycin, and had clinically suspected adriamycin cardiomyopathy or DCM. Histologically, characteristic findings such as cardiomyocyte atrophy, nuclear pleomorphism, and interstitial fibrosis were not observed (A: H-E staining; scale bar, 20 µm; original magnification, ×400). ICDs were visible (B: H-E staining; scale bar, 10 µm; original magnification, ×1000).

On the other hand, immunohistochemistry revealed that there were some area where the intensity of N-cadherin immunostaining was reduced in ICDs, and the other area where the intensity was not reduced. Most of ICDs had a band-like appearance, but some of them had scattering (C–F: N-cadherin immunostaining; scale bar, 5 µm; original magnification, ×1000). Because most of ICDs had a CHF-like morphology, we classified this case as CHF. This case had been treated with adriamycin and may be in the process of transitioning to drug-induced DCM.

Fig. S3


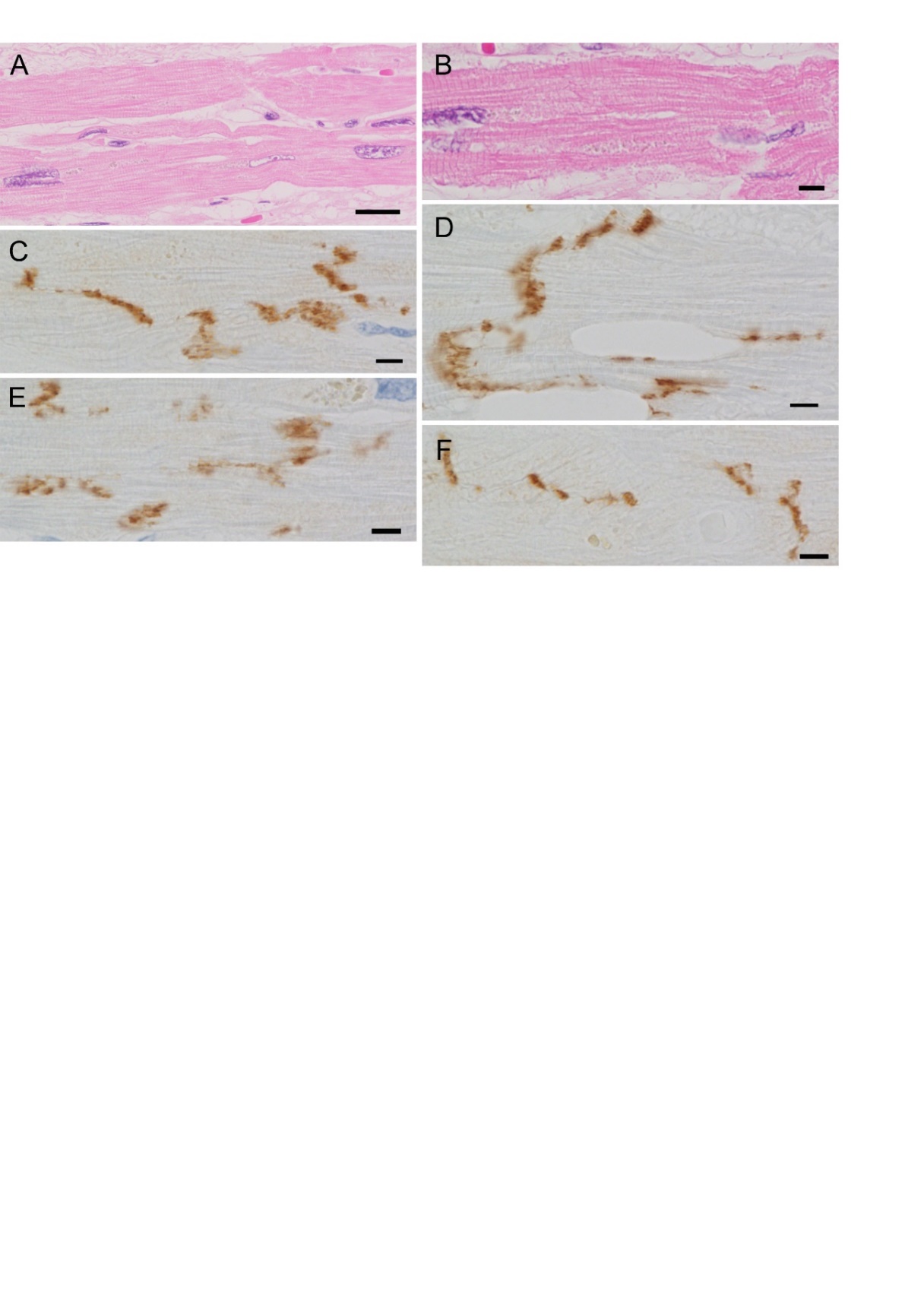


**Fig. S3 A case clinically diagnosed as CHF was considered to be DCM based the results of N-cadherin immunostaining and ICD scattering**

This case was clinically diagnosed as CHF, and an autopsy was performed. Histologically, cardiomyocyte atrophy, nuclear pleomorphism, and interstitial fibrosis were observed (A: H-E staining; scale bar, 20 µm; original magnification, ×400). ICDs were not clearly visible (B: H-E staining; scale bar, 10 µm; original magnification, ×1000).

In immunohistochemistry, N-cadherin immunostaining was reduced in many ICDs, and ICD disintegration was observed (C–F: N-cadherin immunostaining; scale bar, 5 µm; original magnification, ×1000). ICD scattering was apparent. Based on these findings, we have classified this case as DCM.
